# Neutrophil‐T cell crosstalk and the control of the host inflammatory response

**DOI:** 10.1111/imr.13162

**Published:** 2022-11-03

**Authors:** Serena Bert, Suchita Nadkarni, Mauro Perretti

**Affiliations:** ^1^ The William Harvey Research Institute Queen Mary University of London London UK

**Keywords:** autoimmunity, immune‐mediated diseases, immunosuppression, inflammation, pregnancy

## Abstract

While fundamental in their innate role in combating infection and responding to injury, neutrophils are emerging as key modulators of adaptive immune responses. Such functions are attained via both soluble and nonsoluble effectors that enable at least two major downstream outcomes: first, to mediate and control acute inflammatory responses and second, to regulate adaptive immunity and ultimately promoting the development and maintenance of immune tolerance either by releasing immuno‐modulatory factors, including cytokines, or by directly interacting with cells of the adaptive immune system. Herein, we review these novel properties of neutrophils and redefine the pathophysiological functions of these fascinating multi‐tasking cells, exploring the different mechanisms through which neutrophils are able to either enhance and orchestrate T cell pro‐inflammatory responses or inhibit T cell activity to maintain immune tolerance.

## 1 NEUTROPHIL BIOLOGY AND HETEROGENEITY

Neutrophils are the most abundant leukocytes in blood, accounting for 50%–70% of the total circulating population in humans, and serve to perform nonredundant functions as a first line of defense against infection.^1^ For long time, neutrophils have been considered a homogeneous population with the task of detecting foreign organisms or tissue damage to initiate the immune response. However, there is growing evidence that suggests a wider set of functions for neutrophils both in health and disease.^2^ These include a broad array of immunomodulatory functions through which they are able to regulate the activity of innate and adaptive immune cells.^3^

### 1.1 Neutrophils in the inflammatory response

In homeostatic conditions, neutrophils have an average lifespan of 5 days in the blood^4^ and are constantly produced in the bone marrow via granulopoiesis, under the regulation of granulocyte colony‐stimulating factor (G‐CSF), which is involved in the processes of progenitor lineage commitment,^5^ proliferation,^6^ and egression of mature neutrophils from the bone marrow into the bloodstream.^7^ Following inflammatory cues, neutrophil production and release into the circulation are markedly enhanced; these cells then rapidly migrate to the inflamed tissue through a migration process across postcapillary venules, under the guidance of chemoattractant signaling, including complement factors and leukotrienes, as well as the chemokines CXCL8, CXCL1, and CXCL2.^8^ At the site of inflammation, neutrophils cooperate with other leukocytes for pathogen removal through phagocytosis of invading microorganisms, release of cytoplasmic granules, NETosis, activation of the respiratory burst with production of reactive oxygen species (ROS), and release of cytokines for the recruitment of other immune cells.

More recently, neutrophils have been shown to undergo reverse migration, migrating away from inflammatory sites to possibly impact on secondary organ injury (recently reviewed in 9).

A feature of the neutrophil is the complex nature of its cytoplasmic components, in a large part packed into granules and vesicles. These granules are heterogeneous reservoirs of antimicrobial enzymes and proteins, utilized for host defense against pathogens. Classically, they are distinguished based on their contents in azurophil peroxidase‐positive granules, which contain myeloperoxidase, defensins, and serine proteases (eg, cathepsin‐G and neutrophil elastase [NE]), and in peroxidase‐negative granules. The latter are further distinguished in specific granules, enriched in antimicrobial proteins including cathelicidins and lactoferrin as well as the beta‐2 integrin CD11b/CD18, and gelatinase granules, which mainly contain matrix‐degrading enzymes (MMPs) and plasma borne proteins up taken by pinocytosis, like albumin.^10^ Neutrophil granules are central effectors in neutrophil microbicidal activity, but also support cell recruitment and modulate activity of other immune cells. At the site of inflammation, neutrophil granules guide recruitment of inflammatory monocytes through promotion of monocyte activation and extravasation.^11^ Macrophage anti‐microbial activities are also enhanced, including phagocytosis of opsonized bacteria, ROS production, and release of inflammatory cytokines.^12^, ^13^, ^14^ Neutrophil granule contents can modulate adaptive immunity, through functional effects on both lymphocytes^15^, ^16^ and dendritic cells (DC),^17^, ^18^, ^19^ further discussed below.

Neutrophil NETosis consists in the extracellular release of unwound DNA strands in complex with histones and granule proteins, most notably myeloperoxidase (MPO) and NE. Triggered by LPS, phorbol myristate acid (PMA), and CXCL8, the release of NETs is a well‐documented effector function of neutrophils in the elimination of bacteria.^20^ Permanence of these intracellular neutrophil contents at sites of inflammation can also be detected by other cells, especially immune cells and utilized as another method of neutrophil interaction. NETs produced by human neutrophils after in vitro stimulation increase the activation of macrophages,^21^ DCs,^22^ and lymphocytes.^23^ Uptake of NETs by human macrophages and DCs modulates their cytokine profile, with increased production of pro‐inflammatory cytokines, including IL‐1β, IL‐6, and TNF‐α, and augmented the release of chemokines CXCL8, CCL3, and CCL4, most likely involved in subsequent recruitment of other leukocytes.^21^, ^24^ Interestingly though, studies have also reported anti‐inflammatory effects of NET uptake from phagocytes in humans, such as dampening DC activity,^25^ augmented IL‐10 production, and M2 polarization in macrophages,^21^, ^26^ altogether suggesting a multifaceted role of NETs in neutrophil modulation of immune responses.

The neutrophil secretory profile is indeed a key mechanism through which these cells are involved in shaping the inflammatory response at sites of infection. As first recruiters during bacterial infection, neutrophils release a variety of chemokines involved in the recruitment of other leukocytes of both innate and adaptive immunity to the site of inflammation. They are also involved in the production of a broad array of cytokines involved in the modulation of the inflammatory response. Both human and murine neutrophils can produce pro‐inflammatory cytokines TNF‐α, IL‐1β, and other factors, involved in the activation of antimicrobial responses, but also immunoregulatory cytokines like IL‐12, IL‐23, and TGF‐β.^27^ Murine neutrophils are also capable of producing key immunomodulatory cytokines that are either not produced by human neutrophils (eg, IL‐10)^28^ or on which human data still remains controversial (eg, IL‐6, IL‐17, and IFN‐γ).^3^ In any case, neutrophils can produce cytokines themselves or upregulate their release from other effector leukocytes, like macrophages^21^, ^24^, ^29^, ^30^ and DCs,^17^, ^18^, ^24^, ^31^, ^32^ making them key players in regulating the cytokine milieu during inflammatory responses.

The release of neutrophil‐derived particles (extracellular vesicles [EVs], also termed ectosomes or microparticles) ranging in size between 0.1–1.0 μm, has recently become of increasing interest. These heterogeneous particles bear markers specific for their cell of origin are a method for intracellular communication with other immune and nonimmune cells, to transfer a large number of cargo types, including proteins, lipids, and RNA species (mRNA, miRNA).^33^ Produced both in homeostasis and after cell activation, the release of EVs can be used to exert both pro‐inflammatory and anti‐inflammatory immunomodulatory actions. We have shown that human neutrophil EVs are heterogeneous in their protein content depending on the mode of stimulation,^34^ leading us to suggest that neutrophils are geared up to release “tailor‐made” vesicles to reflect, and impact on, their inflammatory surroundings. More recently, Kolonics et al. have confirmed that EVs produced by human neutrophils can have opposing effects depending on the state of neutrophils themselves. EVs produced by resting or apoptotic neutrophils exerted anti‐inflammatory effects, while EVs from activated neutrophils promoted pro‐inflammatory activity in other neutrophils and in endothelial cells.^35^

Altogether this wealth of information identifies, the neutrophil as a polyhedric cell that can regulate a large set of phenomena. While its main, and originally identified, role is to efficiently fight bacteria and other infectious agents, the plethora of factors they can release enables a much wider set of actions that contributes to host well‐being and survival. As such, it has been long known that presence of neutrophils first, and then of dead neutrophils, in inflammatory sites—identified as pus—is indicative of proper resolution of the inflammatory response. This is a feature of the acute inflammatory response which resolves in time and is contained in space, to avoid collateral damage.

On this line, some recent work has indicated that migrated neutrophils, both human cells in in vitro assays and murine cells in vivo models, can govern the resolution of the inflammatory response by crosstalk with other immune cells^29^, ^30^, ^36^ as well as stromal cells.^37^ The end‐point of these properties is often protective and reparative, as recently reviewed.^38^ These pro‐resolving actions of the neutrophil can be attained through multiple mechanisms, one of which is cell death and apoptosis. We discuss below how the neutrophil can influence the behavior of T cells and as such can act as a pivotal player in the adaptive immune arm of the host response.

### 1.2 Neutrophil plasticity

Neutrophils have been long considered a homogeneous population of short‐lived terminally differentiated cells, with a uniform response to host damage and inflammation. In recent years though, accumulating evidence has brought to light a variety of neutrophil phenotypes and functions, and these can be acquired in both physiological and pathological settings, in murine models and human assays. From the presence of neutrophils with different maturation or activation states to the development or expansion of distinct subsets in pathologies, including cancer, sepsis, autoimmune disorders, and chronic inflammatory diseases, neutrophil heterogeneity and functional plasticity have become of interest to unveil the multiple functions that these cells may perform in health and disease.^39^

Neutrophil plasticity in cancer was first highlighted by the presence a neutrophil subset within tumors, denominated tumor‐associated neutrophils (TAN), transcriptionally distinct from normal neutrophils.^40^ To date, TANs have been further shown to acquire distinct phenotypes, leading to the classification into N2, the most predominant and pro‐tumorigenic subset, and N1, a subset that acquires anti‐tumor activity.^41^ The induction of TAN pro‐inflammatory N1 and anti‐inflammatory N2 subsets is dependent on the tumor microenvironment, and specifically, it is linked to TGF‐β promoting the cell differentiation toward ab anti‐tumor N1 neutrophil phenotype,^42^ while IFN‐β favors a pro‐tumor N2 subset^43^ (reviewed in 44). Interestingly, presence of N1 and N2 neutrophil subsets has also been reported in ischemic heart injury in mice: the predominance of each subset in the myocardium was time‐dependent, with an influx of pro‐inflammatory N1 cells immediately postinfarction, followed by a steady influx of anti‐inflammatory N2 cells up to day 7 later, suggesting a reparative role for N2 cells in these settings.^45^

TANs can have a great impact on tumor microenvironment and modulate anti‐cancer immunity. In murine models, TANs dampen adaptive immune responses within the tumor microenvironment through increased expression and activity of Arg I, high production of reactive oxygen species and PD‐L1/PD1 signaling, resulting in suppression of CD8+ T cell and NK cell cytotoxic activity.^46^, ^47^, ^48^ They are also involved in the recruitment of regulatory T cells (Treg) to the tumor by releasing CCL17.^49^ Other TAN subsets instead have the opposite effect and stimulate T cell responses in tumors. A study by Eruslanov et al. identified a predominant subset of TAN displaying an activated phenotype (CD11b^+^CD15^hi^CD66^+^) within early‐stage human lung cancer that were capable of promoting CD4+ and CD8+ T cell proliferation, activation and enhance Th1 cytokine production. These TANs also presented increased production of pro‐inflammatory cytokines and chemokines, which could be involved in leukocyte recruitment and modulation within the tumor microenvironment.^50^

Low‐density neutrophils (LDN) are so termed as they sediment in the PBMC fraction during density gradient centrifugation and have been found in various pathological conditions, most notoriously in cancer, but also in physiological events such as pregnancy. As detailly reviewed by Scapini et al.,^51^ the identification and classification of LDNs has met quite a few controversies over the years due to the absence of specific markers that can uniquely distinguish these cells from classical mature neutrophils. LDNs display neutrophil‐like morphology and express markers of neutrophil lineage, but are heterogeneous in functions, maturation state, and can present different combination of immune markers, indicating possible existence of different LDN subsets. Human LDNs with pro‐inflammatory functions have been observed in autoimmune disorders (ie, systemic lupus erythematosus, rheumatoid arthritis),^52^ where they contribute to secretion of pro‐inflammatory cytokines and advancement of chronic inflammation.^53^, ^54^, ^55^ LDNs with immunosuppressive abilities, also known as granulocytic myeloid‐derived suppressor cells (MDSC) are well renowned for their role in inhibiting anti‐cancer immunity in a wide number of malignancies, but have also been reported in other pathologies in murine models, including cardiovascular disorders,^56^, ^57^ asthma,^58^ neuroinflammatory diseases.^59^ A role for MDSCs has also been found in human pregnancy, where these cells are key players in sustaining fetal tolerance.^60^ The main mechanisms through which MDSCs exert their suppressive function are arginine depletion by arginase I, ROS production, and crosstalk with regulatory T cells (further discussed below).

Different states of neutrophil maturation are linked to neutrophil immunosuppressive or activating functions. A recent study by Marini et al. has reported the use of CD10 for the distinction of mature and immature neutrophils, respectively, with associated suppressive and proinflammatory functions.^61^ As CD10 expression changes during granulocyte differentiation, the Authors were able to distinguish subsets of CD66b^+^CD10^−^ immature neutrophils and CD66b^+^CD10^+^ mature neutrophils within both low‐density neutrophils (LDNs) and normal density neutrophils (NDNs) in healthy G‐CSF‐treated peripheral blood stem cell donors, a finding that was confirmed by nuclear morphology. CD66b^+^CD10^−^ LDNs could promote T cell proliferation, IFN‐γ production and T cell survival, while both CD66b^+^CD10^+^ LDNs and NDNs displayed inhibitory effects on T cells. These functionally distinct neutrophil populations were detected within the blood of solid tumor, lymphoma, and SLE patients in the same study, while other studies have also identified proinflammatory CD10^−^ immature neutrophils in acute myocardial infarction,^62^ in chronic graft versus host disease^63^ and CD10^low^ neutrophils in the synovial fluid of juvenile idiopathic arthritis patients.^64^ Low‐density CD10^−^ neutrophils from metastatic melanoma patients can enhance T cell proliferation in vitro, while CD10^+^ LDNs are suppressive.^65^ Alternatively, Lang et al.^66^ utilized the expression of neutrophil differentiation markers CD11b and CD16 to define different stages of maturation within granulocytic‐derived myeloid‐derived suppressor cells (PMN‐MDSC). These subsets displayed both different nuclear morphologies, in line with their maturation state, and suppressive capabilities: CD11b^+^CD16^+^ PMN‐MDSC had the polymorphonuclear shape of mature cells and the highest suppressive activity on T cells, CD11b^+^CD16^−^, and CD11b^−^CD16^−^ PMN‐MDSCs consisted of banded immature cells with mild suppressive capability.^66^

Splenic neutrophils can provide support to B‐cell functions. Briefly, this novel function is not confined to a single standard cell population. Puga et al. have defined two populations of human B‐helper neutrophils, N_BH1_ and N_BH2_, which differ phenotypically and in their ability to promote immunoglobulin class switching and antibody production by activating B‐cells within the marginal zone of the spleen, via the production of BAFF, APRIL, and IL‐21.^67^ More recently, Deniset et al. have shown that in mice both Ly6G^high^ mature and Ly6G^int^ immature neutrophils co‐exist in the red pulp of the spleen following infection with *S. pneumonia*. As with the N_BH_ neutrophils, these two populations of neutrophils differ in their phenotype and behavior during bacterial clearance.^68^

Distinct neutrophil sub‐populations have also been identified in man, specifically based on different levels of CD62L and CD11b expression and on the expression of the pro‐resolving protein annexin A1 (AnxA1). CD16^bright^CD62L^dim^ neutrophils directly inhibited T‐cell responses during acute LPS systemic inflammation via the integrin Mac‐1, and subsequent release of H_2_O_2_; this subset was further characterized by a hyper‐segmented nuclear morphology.^69^, ^70^ Neutrophil phenotypes can be modulated by drug treatment as we detected in patients affected by giant‐cell arteritis.^71^ During this longitudinal study, a distinct CD16^hi^AnxA1^hi^CD62L^lo^CD11b^lo^ was abundant 1‐week poststeroid treatment, which correlated with disease control by the drug. The lower doses of steroids used at 24 weeks were associated with a CD16^hi^AnxA1^hi^CD62L^hi^CD11b^hi^ phenotype which might correlate with re‐emergence in disease activity including fatal events like stroke.

CD177 (reviewed in 72) is a neutrophil‐specific gene that encodes the neutrophil membrane glycoprotein, Human Neutrophil Antigen‐2a (also known as NB1 or polycythemia rubra vera‐1, PRV‐1). A subset of CD177+ neutrophils has been identified in patients with inflammatory bowel disease: these cells produce IL‐22, leading to enhanced bactericidal activity, thereby limiting intestinal inflammation.^73^, ^74^ It is therefore plausible that a gut‐specific or restricted phenotype of neutrophils may be operative to ensure tight efficacy of the host response.

Altogether, these studies indicate that neutrophils can undergo distinct differentiation routes depending on environmental cues, and as such, these neutrophil subsets can differentially affect disease progression.

## 2 MECHANISMS OF NEUTROPHIL‐T CELL INTERACTIONS

**FIGURE 1:**
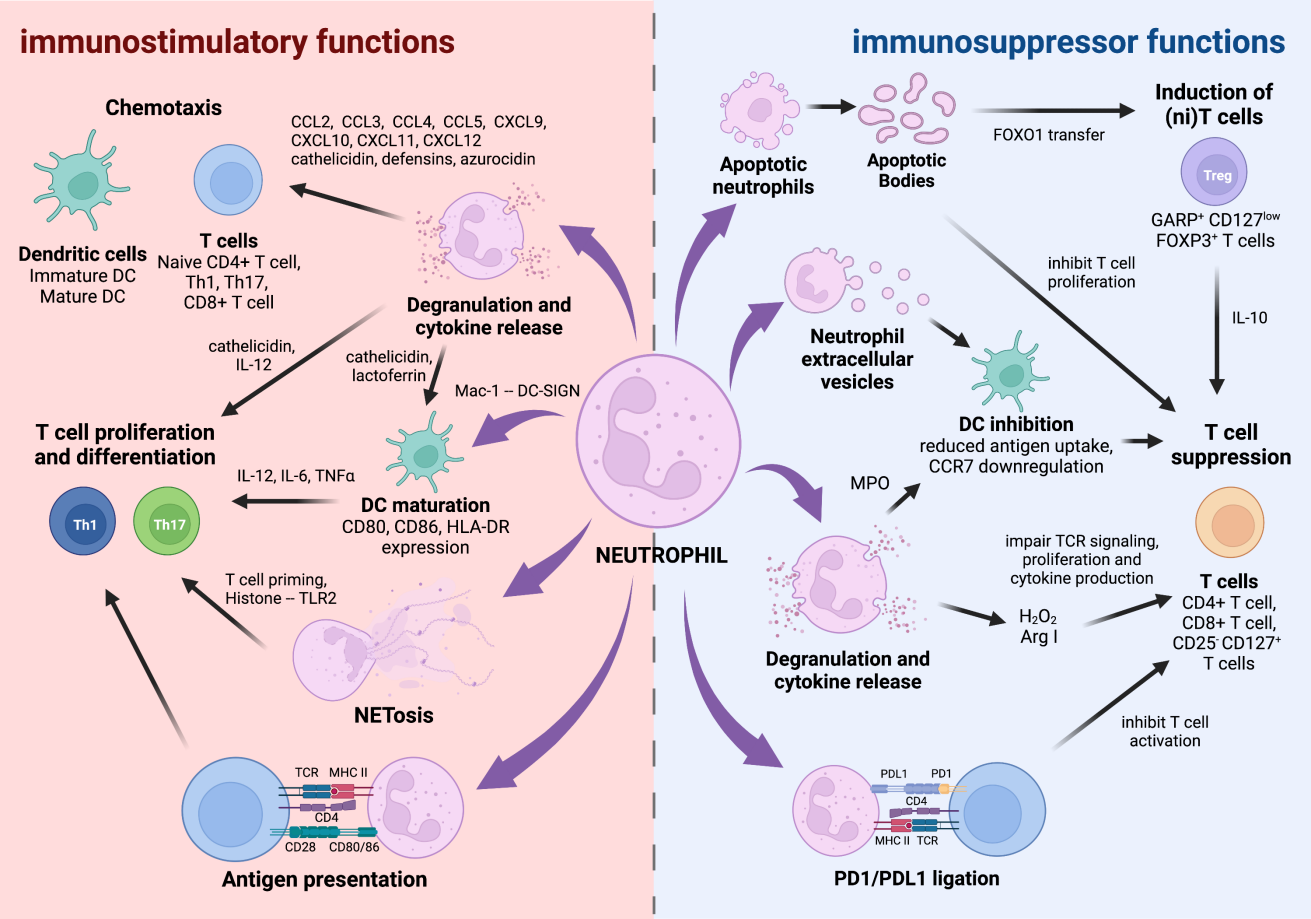
Soluble and insoluble factors of neutrophils regulate the host immune response. Schematic representation of the immune‐regulatory properties of neutrophils to stimulate or inhibit adaptive immune responses. *Left Panel: immuno‐stimulatory functions*. Activated neutrophils can guide T cell and dendritic cell (DC) migration through release of chemokines and granular contents, like polypeptides, during inflammation. Granule contents, NETs, and cytokines released from neutrophils promote T cell proliferation and differentiation into Th1 and Th17 subsets, through direct interaction with T cells or through modulation of DC activity. Neutrophils can also acquire antigen‐presenting capabilities to support T cell activation during inflammatory responses. *Right Panel: immuno‐suppressor functions*. On the other hand, neutrophils can inhibit T cell and DC responses, supporting resolution of inflammation and immune tolerance. Neutrophil apoptotic bodies dampen pro‐inflammatory T cell responses through induction of regulatory T cell‐like niT cells and suppression of T cell proliferation, while neutrophil extracellular vesicles inhibit activity of DCs. The release of reactive oxygen species (eg, H_2_O_2_) and granule proteins Arg I and MPO are also used for both T cell and DC immunosuppression. Lastly, neutrophils can inhibit T cell activation through cell contact mechanisms, via upregulation of PD‐L1 and PD1 ligation. *Created with BioRender™*

While fundamental in their innate role in combating infection and responding to injury, neutrophils are emerging as key modulators of adaptive immune responses. Such functions are attained via both soluble and nonsoluble effectors that enable at least two major downstream outcomes: first, to mediate and control acute inflammatory responses and second to regulate adaptive immunity and ultimately promote development and maintenance of immune tolerance either by releasing immuno‐modulatory factors, including cytokines, or by directly interacting with cells of the adaptive immune system (Figure 1).

### 2.1 Neutrophil effector functions in orchestrating T cell responses

Well‐known to be involved in the recruitment and activation of monocytes and macrophages during infection,^11^, ^18^, ^75^ neutrophils have become of great interest for their multiple roles in modulating both T cell and DC activity, becoming key players in bridging innate and adaptive immune responses. Activated neutrophils initiate a series of effector functions, from the release of granules and intracellular material (ie, NETs) to production of cytokines and chemokines, which are liberated in the local microenvironment to coordinate recruitment, activation, and effector function of adaptive immune cells. Human neutrophil granule peptides possess T cell chemotactic properties^76^, ^77^ and can directly promote migration of naive T cells and immature dendritic cells^78^ (Figure 1), possibly guiding adaptive immune cells to sites of microbial infection where their activation can be triggered. Upon activation, both human and mouse neutrophils can release a broad array of cytokines and chemokines,^27^ which have been indicated to play a role in both recruitment of different T cell subsets and in promoting polarization of newly activated T cells. Pelletier et al. and Gasperini et al., for example, reported that human neutrophils were able to chemoattract both Th17 cells and Th1 cells after stimulation with LPS and IFN‐γ through release, respectively, of the chemokines CCL2 and CCL20^79^ and CXCL9, CXCL10 and CXCL11.^79^, ^80^ In mice, both bacterial and parasitic infections also promoted the release of neutrophil‐derived chemokines for the recruitment of DCs, including CCL3, CCL4, and CCL5.^31^, ^81^ Neutrophils can induce CD8+ T cell migration as well in both mouse and human,^78^, ^82^ with a study by Lim et al. describing a novel mechanism of neutrophil‐mediated T cell recruitment in a mouse model of influenza infection.^83^ In these settings, neutrophils guide CD8+ T cell migration after infection by leaving a trail of membrane particles enriched in CXCL12. These vesicles act as a chemotactic signal for the recruitment of effector CD8+ T cells to the trachea and are essential for promoting virus clearance.

As demonstrated by the in vitro studies of Minns et al., the modulatory effects of neutrophils on T cells can vary greatly not only depending on the state of the T cells but also on the activation state of neutrophils.^84^ This results in a broad range of outcomes downstream neutrophil‐T cell interactions, making the neutrophil pivotal for the regulation of adaptive immune responses. Generally, activated neutrophils have been observed both in humans and mice, in vitro and in vivo, to promote T cell activation, proliferation, and differentiation to T‐helper cell subsets (ie, Th1, Th17) and effector CD8+ T cells, which would favor adaptive immune responses at the site of inflammation.^82^, ^84^, ^85^, ^86^

Neutrophil activation leads to the release of antimicrobial peptides and cytokines, which not only mediate neutrophil effector functions but also act as cellular signaling molecules to both innate and adaptive immune cells. Through these mediators, neutrophils can promote T cell activation and differentiation via direct interaction and modulation of DCs (Figure 1). The neutrophil granule peptide cathelicidin (LL‐37 in humans, mCRAMP in mice) has been reported to have immunomodulatory effects on both T cells and DCs, regulating Th1 and Th17 differentiation.^15^, ^17^ A study by Minns et al. found that cathelicidin released by neutrophils acted as a potentiator of Th17 responses by T cells both in vitro and in vivo, in humans and mice.^15^ They observed that neutrophil‐derived cathelicidin induced expression of the Th17 transcription factor RORγt and production of IL‐17A and IL‐21 in CD4+ T cells with a concomitant decrease in Th1‐associated genes, but required Th17‐promoting conditions, most importantly TGF‐β1 signaling. On the other hand, cathelicidin can also have Th1‐inducing properties through the modulation of DC cytokine profile. In the presence of LL‐37, human DCs increased expression of IL‐12 while decreasing expression of IL‐4, which are required for Th1 and Th2 differentiation, respectively. Consequentially, DCs could stimulate the production of IFN‐γ in T cells, a marker of Th1 polarization.^17^ Therefore, neutrophil cathelicidin can both have direct effects in guiding T cell polarization when exposed to the correct environmental cues or indirectly influence T cell differentiation by modulating the polarizing environment itself.

Aside from modulating DC cytokine production, neutrophils can also directly promote Th1‐ conditions through their own production of IL‐12, as neutrophils possessing stores of IL‐12 have been found to accumulate at the site of inflammation in a murine model of Toxoplasma gondii infection.^87^ Th1 immunity is also enhanced during cytomegalovirus (CMV) infection, where Fraccarollo et al. identified a population of immature CD66b^+^CD16^+^CD10^−^ neutrophils that enhanced production of IL‐12 and IFN‐γ by CD4+ T cells in CMV‐seropositive patients. Transwell™ coculture experiments indicated that this modulation occurs in a cell contact‐independent way, but the soluble mediators responsible have not yet been identified.^62^ Th1‐promoting ability of neutrophils in response to infection has been observed in a murine model of pneumonia as well, where neutrophils were found to be required for successful Th1 polarization and neutrophil depletion‐induced T cells to switch to a Th2 phenotype.^88^

Pathogen infection provokes release of NETs from activated neutrophils, which aside from their antimicrobial properties can also be used to interact with other immune cells, of which T cells and DCs are primary cellular recipients (Figure 1). Human NETs can prime T cells through direct contact and lower their activation threshold, thus stimulating their activation and response to antigens.^23^ More recently, a study by Wilson et al. showed that NETs can not only directly stimulate T cell activation, but also guide their polarization in mice.^89^ Histone interaction with TLR2 on the surface of CD4+ T cells led to phosphorylation of STAT3 with subsequent induction of expression of RORγt, the key transcription factor of Th17 cells. In the presence of Th17‐promoting cytokines IL‐6 and TGF‐β, this enhanced the production of IL‐17 and expression of Th17‐associated genes in CD4+ T cells.

Aside from antimicrobial effector functions, neutrophils may also possess other mechanism through which they can influence T cell activity. An example of this is a novel study by Dunsmore et al. investigating neutrophil‐T cell interactions in HIV patients. They found that activated pro‐inflammatory neutrophils, such as the ones found in HIV patients, shed Galectin‐9 (Gal‐9) from their surface, leading to high plasma Gal‐9. Neutrophil‐derived Gal‐9 binds to CD44 on T cells and is able to enhance activation of both CD4+ and CD8+ T cells, measured by increased expression of HLA‐DR and CD38.^90^

Modulation of DC activity is also an indirect pathway through which neutrophils can affect the ensuing adaptive immune response within the inflamed tissue. Activated neutrophils can induce DC maturation through direct interactions via the integrin Mac‐1 and the C‐type lectin, DC‐SIGN in both mice and humans,^91^ a key prerequisite for the induction of effector T‐cell responses. They can also enhance expression of co‐stimulatory molecules, including CD80, CD86, and HLA‐DR on human and mouse DCs upon exposure to pathogens^18^, ^31^, ^32^, ^92^ and can direct DC cytokine profile, in turn modulating T cell polarizing conditions^17^, ^18^, ^31^, ^32^ (Figure 1).

In inflammatory settings, different studies have indicated that neutrophils could also stimulate CD4+ and CD8+ T cell activation by acting as conventional antigen‐presenting cells (Figure 1). Both human and mouse neutrophils express co‐stimulatory molecules CD80, CD86, MHC‐II, and MHC‐I after stimulation by TLR ligands, as well as the DC marker CD83.^85^, ^86^, ^93^, ^94^, ^95^ As these molecules are usually not detected on the surface of resting neutrophils, Sandiland et al. investigated whether there are intracellular reserves which can readily translocate to the cell surface when required. Indeed, this study showed that during human neutrophil activation, cross‐linking of Mac‐1 leads to rapid translocation onto the plasma membrane of CD80, CD86, and MHC‐II, from their cytoplasmic storage in secretory vesicles.^93^ Thus, in contrast to classical antigen‐presenting cells, which constantly present detectable levels of these molecules on their surface, neutrophils can be seen as a flexible antigen‐presenting cell that may act to reinforce antigen presentation when needed. In mice, neutrophils were in fact shown to use MHC‐II and MHC‐I to present antigens, respectively, to CD4+ and CD8+ T cells and even direct CD4+ T cell polarization towards Th1 and Th17 phenotype^85^ and promote CD8+ T cell differentiation to effector cells.^86^ Although these novel antigen‐presenting neutrophils display markers of neutrophil lineage (ie, CD66 in human neutrophils), they undergo morphological and functional changes to express markers typical of DCs.^93^, ^95^ This might suggest development of a distinct subset of neutrophils to perform antigen‐presenting functions along more canonical antigen‐presenting cells during both pathogen infection and chronic inflammation. More recently, Mysore et al. have reported that mature neutrophils can convert into antigen‐presenting cells (nAPC) after engagement of FcγRIIIB and endocytosis of anti‐FcγRIIIB‐antigen complexes in mice. As discussed above, these nAPCs express DC markers like CD11c, CD80, CD86, MHC‐II, CCR7 and present changes in nuclear morphology, yet they still maintain some neutrophil functions. Most importantly, nAPCs induced CD4+ and CD8+ T cell responses just as efficiently as DCs and this feature could be harnessed for anti‐tumor immunity in vivo.^96^

## 3 NEUTROPHILS AND T CELL SUPPRESSION

Neutrophils have the potential for promotion of T cell activation and differentiation, but also for inhibition of T cell response (Figure 1). The mechanisms dictating this dichotomy of end‐responses are not yet fully understood but have been suggested to be influenced by different activation states of neutrophils and T cells during their interaction, as well as by modulation of neutrophil‐T cell crosstalk outcome by soluble mediators present in the local microenvironment. Furthermore, this inter‐cellular modulation can regulate the interaction of T cells with different neutrophil subsets with predominant suppressive functions (ie, G‐MDSCs).

In their in vitro study, Minns et al. observed that while TNF and LPS‐primed human neutrophils promoted T cell activation, in the absence of any pathogen or inflammatory stimulus, resting neutrophils had the ability to suppress the early activation of T cells.^84^ Based on their observations, they hypothesized that the different outcome on T cell activity during neutrophil‐T cell crosstalk might be influenced by the state of both neutrophils and T cells. In line with this hypothesis, our own studies have indicated that different neutrophil activation states could affect their crosstalk with T cells during pregnancy.^97^ Exposure to the normal maternal hormonal milieu induced a CD16^lo^CD62L^lo^CD11b^lo^AnxA1^hi^ quiescent phenotype in human neutrophils, which gained the ability to promote T cell differentiation into GARP^+^CD127^lo^Foxp3^+^ regulatory T cells, termed neutrophil‐induced T cells or niT cells (Figure 1). Our data indicate that niT cells are essential for the establishment of fetal tolerance during gestation. During pregnancy complications such as preeclampsia, neutrophils are activated and display a pro‐inflammatory phenotype with high levels of CD16, CD62L, and CD11b, low AnxA1, and lost their ability to induce niT cells.^97^

On the other hand, specific effector functions and soluble mediators produced by activated neutrophils have been amply described to suppress T cell responses, most notably the production of ROS and arginine metabolism by neutrophil‐derived Arg I (Figure 1). Early studies showed that activated neutrophils from cancer patients suppress T‐cell function through release of H_2_O_2._ ^98^ This suppressive capacity of neutrophils on T‐cells occurs in humans during LPS‐induced acute inflammation, dependent on bacterial opsonization through neutrophil Mac‐1 (integrin α_M_β_2_; CD11b/CD18), and subsequent H_2_O_2_ release leading to suppression of T‐cell proliferation.^4^ In humans, neutrophil‐derived ROS impair T cell activation by suppressing TCR signaling by interfering with PLC‐γ1 phosphorylation and Ca^2+^ mobilization,^99^ and inhibiting T cell cytokine production.^98^ Exposure to high doses of ROS can also cause apoptosis or necrosis of T cells.^100^ Interestingly, in vitro cultures of human T cells with exogenous H_2_O_2_ of granulocytes showed that regulatory T cells present greater resistance to ROS‐induced cell death than conventional CD4+ T cells. This would favor synergy between Treg and neutrophil suppressor functions in tumors, where both cells are key players in inhibition of anti‐tumoral immunity.^101^ Impaired neutrophil ROS production has been observed in the synovial fluid of patients with juvenile idiopathic arthritis, which become unable to suppress T cells thus contributing to prolonged inflammation in the joints.^64^

The mechanisms of action of Arg I on T cell suppression have become of interest as a possible novel target for the modulation of T cell responses. Arg I is stored within gelatinase granules and released into the extracellular environment by degranulating human neutrophils.^102^ This enzyme depletes arginine in the surroundings, which is the cause of inhibition of T cell proliferation due to early arrest of cell cycle progression, downregulation of TCR‐ζ chain and reduced IFN‐γ production.^16^, ^103^ Arginine depletion impairs dephosphorylation of cofilin in human T cells, resulting in an impaired TCR signal transduction and immunological synapse formation between T cells and the antigen‐presenting cell.^104^ Most importantly, these effects are readily reversed through inhibition of Arg I. A recent study by Vonwirth et al. showed that human neutrophil treatment with Arg I inhibitors not only prevented T cell suppression, but instead it led to T cell hyperresponsiveness,^105^ monitored as increased proliferation, cytokine secretion, re‐expression of co‐stimulatory molecules and cytotoxicity. These outcomes were reproduced utilizing T cells and neutrophils from the blood of multiple myeloma patients, highlighting a therapeutic potential for Arg I inhibition in enhancing anti‐cancer T cell responses and evading PMN‐mediated immunosuppression.

MPO is a major granule protein in neutrophils which can display immunosuppressor function.: Odobasic et al. demonstrated that neutrophil‐derived MPO inhibits T cell responses through modulation of DCs in murine models of antigen‐induced arthritis and delayed‐type hypersensitivity. These effects include interference with the uptake of antigen by DCs and downregulation of CCR7 expression, thereby limiting DC migration to lymph nodes. Neutrophil‐mediated DC modulation resulted in dulled T‐cell responses, ultimately limiting tissue damage^19^ (Figure 1).

The PD‐1/PD‐L1 pathway is another key mechanism for the regulation of T cell immunity via neutrophils: binding of PD‐1 on T‐cells to the inhibitory ligand PD‐L1 expressed by a variety of cells suppresses T‐cell proliferation and cytokine production^106^ (Figure 1). IFN‐γ, which is produced during local and systemic inflammation, upregulates PD‐L1 on human neutrophils, allowing direct T‐cell suppression via PD‐1.^107^ Although such neutrophil‐mediated PD‐L1/PD‐1 immune suppression is beneficial in bacterial infection,^108^ it may be detrimental in pathologies where immune suppression worsens disease outcome including sepsis,^109^ HIV,^110^ and cancer.^111^

We have mentioned before the multiple functions of neutrophil EVs. These microstructures released by activated neutrophils can be taken up by phagocytes to regulate their activity and promoting resolution of inflammation. Studies by Eken and Gasser et al. have shown that human neutrophil EVs can dampen the inflammatory response of macrophages, inducing a transcriptional reprogramming toward a pro‐resolving and reparative phenotype by inducing release of TGF‐β1^29^ and downregulating pro‐inflammatory cytokine production.^30^ In DCs, the uptake of human neutrophil EVs inhibited their maturation, reducing phagocytic activity and expression of CCR7. As seen with macrophages, neutrophil EV‐treated DCs showed low cytokine production but increased release of TGF‐β1. Consequentially, these DCs were unable to stimulate T cell proliferation.^112^ The inhibition of proinflammatory functions by human neutrophil EVs can also involve activation of the MerTK pathway and blockade of NF‐κB in macrophages,^30^ but a similar mechanism was observed in DCs when exposed to apoptotic cells in mice as well.^113^

Neutrophils can in fact also release a type of large vesicle during apoptosis, termed apoptotic body.^114^ These vesicles have also been studied in the context of suppression of immune responses and induction of resolution of inflammation through interactions with both innate and adaptive immune cells (Figure 1). Shen et al. demonstrated that human neutrophil apoptotic bodies suppress resting CD25^−^CD127^+^ T‐cell proliferation, by impeding phosphorylation of STAT5, and subsequent IL‐2 production.^115^ More recently, our own work revealed that human neutrophil exposure to the pregnancy hormones progesterone and estriol induces an interesting phenotype with potent roles on T‐cell activation. Short incubation periods of neutrophils with physiological concentrations of estrogen promote AnxA1^hi^CD62L^lo^CD11b^lo^ phenotype, endowed with anti‐inflammatory actions.^116^ An interesting discovery was made when cells exposed to these hormones became apoptotic since this subpopulation induced formation of CD4^+^ T‐cells bearing a GARP^+^CD127^lo^FOXP3^+^ phenotype following antigen activation. These niT cells produce IL‐10, IL‐17, and VEGF and promote vessel growth in vitro. Mechanistically, the T cell phenotype arises as a result of transfer of the transcription factor FOXO1 from the neutrophil to the T‐cell via apoptotic bodies: in vitro, incubations of >16 h were required to promote niT cells, allowing apoptosis of the cultured neutrophils. This novel path for neutrophil‐T cell interaction was then translated in vivo, linking it to pregnancy outcome. Thus, we observed that neutrophil depletion during murine pregnancy leads to abnormal development of the fetal‐maternal unit and reduced embryo development. Abnormalities in the placental architecture were noted, with poor trophoblast invasion and spiral artery development in the decidua, accompanied by significantly attenuated niT cell numbers in draining lymph nodes. Pro‐apoptotic neutrophils, and subsequent niT cell induction, are deficient in women suffering from placental pregnancy complication, for example, pre‐eclampsia, thereby establishing a novel paradigm for neutrophils and neutrophil‐derived apoptotic bodies in maintaining maternal tolerance.^117^ It is not fully defined, as yet, where neutrophils encounter T cells in vivo, and whether the site of interaction may change between physiological and pathological settings. However, it is tempting to speculate that neutrophils are able to orchestrate more than just the cellular response but also to ensure tissue homeostasis too, and the different outcome will vary with their activation status and their environment.

Inhibition of T cell activation and proinflammatory responses is also mediated by specific neutrophil subsets, the most noteworthy of which are granulocytic myeloid‐derived suppressor cells (G‐MDSCs). G‐MDSCs are characterized by high expression and activity of Arg I in both human and mice, which hinders T cell activation and expansion.^118^ In mice, CD11b^+^Ly6G^+^Ly6C^low^ G‐MDSCs acquire higher arginase and MPO activity, as well as increased ROS production in tumor‐bearing mice compared to CD11b^+^Ly6G^+^Ly6C^low^ neutrophils from naive tumor‐free mice, which mediates tumor immunosuppression.^119^ ROS production is in fact another key component of G‐MDSC biology. As described by Ohl et al., ROS are not only important effectors for immunosuppression, but also mediate the activation of G‐MDSC transcriptional programs, inducing transcriptional and metabolic reprogramming in these cells and maintain their identity as immature cells by blocking their differentiation.^120^ In mice, G‐MDSCs were also shown to utilize nitric oxide‐related pathways to suppress T cell responses, increasing their expression of NADPH oxidase 2 (gp91^phox^) and endothelial nitric oxide synthase (eNOS) to produce peroxy‐nitrites (PNT).^121^ Elevated PD‐L1 on G‐MDSC has been reported in many different neoplasms as well, contributing to tumor escape from anti‐cancer T cell responses.^122^, ^123^, ^124^

Lastly, G‐MDSCs can further support inhibition of adaptive immune responses through their crosstalk with regulatory T cells (Treg). MDSCs and T cells represent major cellular components within the tumor microenvironment and can cooperate to suppress anti‐tumoral immunity through different mechanisms that have been described and summarized in a recent review by Haist et al.^125^ Among the mechanisms reported are the induction of Treg recruitment, differentiation, and proliferation by MDSCs. Simultaneously, cytokines produced by Tregs can also promote MDSC maintenance and immunosuppressive activity. The crosstalk between these two immune cell subsets occurs through both soluble mediators (eg, TGF‐β) and cell–cell interactions and establishes a positive feedback loop that efficiently maintains immunosuppression in tumors. Aside from tumor immune evasion, a setting in which G‐MDSCs are of particular interest is pregnancy. In fact, increased levels of G‐MDSCs can be found in the blood and placenta during gestation, where they are involved in the shift toward Th2 polarization, induction, and expansion of Tregs and suppression of Th1 immune responses, which we have previously explored in a review elsewhere.^126^

### 3.1 Sites of neutrophil‐T cell in the lymph nodes

The vast majority of mechanisms of neutrophil‐T cell interactions has been deciphered by utilizing in vitro culture systems, leaving open the question of where these interactions may actually occur. Lymph nodes represent a pivotal meeting point for activation and regulation of T cells, which has brought forth the question of whether neutrophils are able to enter lymph nodes draining inflammation sites to carry out their immunomodulatory roles. Indeed, recent studies have reported localization of neutrophils within these lymphoid organs, although the full extent of how neutrophils migrate to these sites and how they interact with T cells is still being investigated.

Migration of neutrophils to draining lymph nodes occurs both during infection and in sterile inflammation, and is characterized by unique dynamics: using two‐photon scanning laser microscopy, Chtanova et al. reported that during mouse Toxoplasma gondii infection, re‐localization of neutrophils into an infected lymph node begins with the migration of individual cells, followed by further neutrophil influx from distal sites, culminating into a “swarm” of large numbers neutrophils that cluster at the sub‐capsular space of the infected lymph node.^127^ Such a coordinated “attack” within the lymph node seems specific to neutrophils. Neutrophils are also capable of migrating to draining lymph nodes via the lymphatic vessels. In mice infected with *Mycobacterium bovis*, neutrophils act as bacterial shuttles, transporting bacilli from the skin to the draining lymph nodes via lymphatic migration and directly making contact with DC or with T‐cells, indicating that in such instances, neutrophils could deliver antigen to DC for further processing, or act as antigen‐presenting cell themselves.^128^ The homing receptors required for this path of migration differ from those required for migration via HEVs, and the route of access to the lymph nodes (ie, HEV or lymphatics) appears to be dictated by the type of stimulation used. For example, while CCR7 was shown to be important for mouse neutrophil migration to the draining lymph nodes following stimulation with complete Freund's adjuvant,^129^ it is dispensable for migration via the lymphatics following bacterial infection or stimulation with immune complexes, instead relying on CXCR4.^130^, ^131^ Recently, neutrophil localization within mouse lymph nodes was further investigated, showing that resting or in vitro‐activated neutrophils primarily localized within the subcapsular sinus and the medullary region of the lymph nodes, but do not migrate into the parenchyma. However, in vivo infection with heat‐inactivated *P. aeruginosa* lead to neutrophil infiltration B cell follicle and T cell zone of popliteal lymph nodes, indicating a possible generation of selective neutrophil chemoattractants within the inflamed lymph node to guide these cells into the parenchyma. Neutrophils co‐injected with DCs also displayed some migration within the parenchyma, suggesting that DCs might support neutrophil migration.^132^ Presence of neutrophils in the lymph nodes has also been detected during the resolution of inflammation, during which a population of phagocytically active neutrophils was found to transport zymosan to the lymph node cortex in a mouse model of resolving peritonitis.^36^

## 4 CONCLUDING REMARKS: OUTLOOK ON NEUTROPHIL‐T CELL CROSSTALK IN DISEASE

The conventional view on the biological function of neutrophils is rapidly changing and offering, therefore, new opportunities for a better understanding of disease pathogenesis and the development of innovative therapeutic approaches.

The ability of neutrophils to modulate adaptive immunity by both enhancing T cell responses during antimicrobial and inflammatory responses and inhibiting T cell activity when no longer required is indicative of a complex and multi‐layered immune‐regulatory activity exerted by these cells, and this occurs through various mechanisms (Figure 1). These dichotomous functions have also led to the challenging of the notion of neutrophils simply as villains, hence unwanted, in various pathological tissues. Neutrophils can indeed be drivers of inflammation through their activity and by harnessing adaptive immunity. As discussed, neutrophil crosstalk with Th17 cells has been indicated to play a major role in the pathogenesis of inflammatory bowel diseases, including reciprocal recruitment, activation, and promotion of Th17 polarization.^133^ On the other hand, the emerging immuno‐regulatory and tissue‐protective roles of neutrophils have highlighted a role for these cells to resolve inflammation as well. As an example, two studies have recently explored the use of immunosuppressive G‐MDSC‐derived exosomes in murine models of collagen‐induced arthritis^134^ and sodium dodecyl sulfate‐induced colitis.^135^ In both cases, disease severity was greatly attenuated by exosome treatment through inhibition of T cell responses and suppression of Th1 and Th17 immunity. In colitis, G‐MDSC exosome treatment also promoted Treg expansion and accumulation in the lymph nodes.

Although enhancement of neutrophil immunosuppressive functions could possibly be beneficial in autoimmunity and chronic inflammatory diseases, inhibition of these functions has become of great interest in cancer. Inhibitors of arginase I have shown potential as possible potentiators of antitumoral T cell responses thus helping to prevent tumor evasion of immunosurveillance.^105^

The wide range of proinflammatory and immunoregulatory functions that have been attributed to neutrophils have been described in both murine models and in humans, but many mechanisms derive mainly from studies conducted in vitro. Worthy of consideration in this scenario is the difficulty of obtaining highly pure neutrophil cultures. Pelletier et al.^136^ have in fact observed that presence of contaminating leukocytes in human neutrophil cultures isolated through the most frequently used gradient centrifugation method could produce significantly different results compared to highly pure neutrophil cultures isolated via immunomagnetic‐negative selection. The contrasting results on the ability of resting neutrophils to activate T cells in specific in vitro systems highlight the need to apply rigorous purification protocols to remove contaminating cells in order to reduce, if not eliminate, discrepancies between studies on neutrophil functions.^136^

In conclusion, the textbook notion that neutrophils are short‐lived, fast‐acting immune cells, and their role is exhausted after the early phase of the host response has been markedly re‐evaluated: neutrophils remain nonredundant cellular effectors of the first response to infection and xenobiotics, but are also endowed with several other roles. Therefore, their biological significance is much more extended and their impact on the whole of the immune response, innate and adaptive, is now emerging with consistency. As discussed in this review, such a broad spectrum of effects downstream neutrophil recruitment and/or activation is due to the large production of soluble and insoluble mediators that these cells can release. While soluble mediators like ROS and leukotriene B_4_ have been studied since the '80 s, the ability of neutrophils to also synthesize and release cytokines has been pioneered by Cassatella and colleagues.^137^, ^138^ As cytokines exert multiple actions, this observation has rapidly broadened the biological properties promoted by neutrophils from the mid‐90s onward. The current view on the fundamental role that neutrophils exert on all arms of the host response is, at least in part, consequent to the discovery that these cells can deliver also insoluble messages to produce downstream long‐lasting effects. The work discussed here on NETs and EVs goes along this line of thought. These complex microstructures, filamentous or globular, can present to target cells several mediators and factors at the same time, and this “multiple presentation” can elicit more complex downstream effects, potentially due to synergistic or complementary actions. Herein, we have focused on the neutrophil‐T cell crosstalk and discussed how neutrophil‐derived soluble and insoluble effectors can impact on T cell proliferation and differentiation. We propose that detailed definition of this *inter‐cellular affair* can offer opportunities for therapeutic intervention, predicting that if this is achieved, the therapeutic benefit will be long‐lasting due to the reprogramming of the T cell afforded by neutrophil‐derived molecules and structures. Time will tell if this prediction will be fruitful for the millions of patients affected by immuno‐mediated diseases.

## CONFLICT OF INTEREST

The Authors have no conflict of interest to declare.

## Data Availability

Data sharing is not applicable to this article as no datasets were generated or analyzed during the current study.
